# Usage of sorbents for diminishing the negative impact of substances leaking into the environment in car accidents

**DOI:** 10.3389/fpubh.2022.957090

**Published:** 2022-09-16

**Authors:** Iveta Marková, Jozef Kubás, Katarína Buganová, Jozef Ristvej

**Affiliations:** ^1^Department of Fire Engineering, Faculty of Security Engineering, University of Žilina, Žilina, Slovakia; ^2^Department of Crisis Management, Faculty of Security Engineering, University of Žilina, Žilina, Slovakia

**Keywords:** risk, dangerous substance leakage, environmental impact, car accident, sorbents, safety, crisis management, civil protection

## Abstract

**Background:**

Car accidents are often accompanied by dangerous substances leaking into the environment. A proper reaction to the leaking substances, utilizing appropriate sorbents, is necessary for diminishing the negative impact of such events. Sorbents as substances of initial intervention in car accidents (as well as industrial and ecological accidents) are a crucial tool for solving crises connected with dangerous substances escaping into the environment. The risk resulting from the given realities is described in detail in the introduction of the article.

**The goal:**

The goal is describing elements of crisis management in dangerous substance leakage and an analysis of sorption resources for quick and efficient interception of leaking substances, water, ethanol, oil, and gasoline in particular, as a reaction to such events.

**Methods:**

The quality of a sorption resource is determined by a parameter called the sorption capacity, which has been established according to the ASTM F716-18 standard. Loose nature-based sorbents (peat) and synthetic silicate-based SiO_2_, Al_2_O_3_, Fe_2_O_3_, and polypropylene-based ones were observed. The research has been realized on a water, oil, gasoline, and ethanol sorbate. Each experiment was repeated three times

**The results:**

The results attest to the diversity of sorption capacity in comparing nature-based, silicate-based, and polypropylene-based sorption materials. The highest sorption capacity values were reached with the Sorb 4 sample, which is based on 66% of silica and 18% of alumina. The stated ratio is important, because the Sorb 3 sample contains 85% of silica and 6% of alumina and its absorption capacity values are significantly lower.

## Introduction

Risk has become a deciding part of the societal and economic environment and has influenced a wide spectrum of organizations in all industries. Every organization which aims to survive, develop and be sustainable, must be prepared to face all challenges that today's uncertain age presents. Fast technical and technological tempo of progress, dramatic and ever-changing conditions in all walks of life and general uncertainty have all incited enterprises to look for early and adequate solutions to their persistent problems and incidents, as to not allow them to grow into a crisis (1–3).

Modern society brings with it many evens which have a significant impact on the lives of citizens and on the environment. The advancing industry, automation and the increasing need for travel also has its negatives. Among these negatives also number car accidents, which are often accompanied by the leakage of dangerous substances into the environment. To mitigate the negative impact of such an event, a correct reaction to the escaping substances utilizing sorbents is needed. Choosing sorbents is often more difficult than it initially seems. Their manufacturers do provide the properties of individual substances, but in practice various deviations may occur.

Taking up risks (RT) is a part of the decision process in situations, which include uncertainty and in which the probability of every outcome is already evident – rewards and/or negative impacts (4–6). The persons who subject themselves to risk have a tendency of making decisions with high potential rewards and high potential unfavorable results, which can depend on biased perception and susceptibility. It is crucial to effectively communicate with people about different types and effects of risks they face, and about the corresponding measures they should accept according to the given information (7, 8). Differences in individual character traits of responsible persons affect risk susceptibility because they include motivational forces which support risky decisions, isolate from the fears of negative repercussions, and serve as cognitive barriers (9).

The majority of accidents including unregulated reactions is associated with the failure of monitoring and safety measures, or with human error. Throughout the years the attention has shifted from explaining how the accident occurred, through proposing and implementation of protection measures, to prevention utilizing inherent safer elements and to reassessment of processes, solving questions of safety, health, and environmental protection (10, 11). Learning from previous failures is one of the pillars of the modern approach to risk management: the end goal of industrial accident analysis is generating acquired knowledge, so that a repeated occurrence of the accident can be prevented; Events that have the potential to create dangerous situations, however, can also contribute to learning and memory (12).

The approach to evaluating environmental risks is based on a process of evaluating societal risks. In today's day and age, however, a unified approach to evaluating environmental risks is not firmly defined, since it is a unified methodical approach to evaluating said risk. The procedure leans on the principle of determining the acceptability of risk based on the ratio of the frequency of such an event occurring and the fatal consequences of the event. The main difference lies in the assessed subject. Simply said, even though the object of the evaluation is a human, as the only biological species in the case of societal risk, we are convinced of the need to evaluate the probability of plants, animals and parts of the environment being unfavorably affected, in real ecosystems of individual components in the case of environmental risks (13–15).

In accordance with a detailed evaluation of risks, vulnerability analyses (calculation, quantification of relevant damages) with respect to the probability of the analyzed unfavorable event occurring must be applied. Index methods unfortunately do not offer a holistic view of the risk. They usually stem from prior analyses of risks and must be supplemented by a subsequent quantitative evaluation of environmental risks (16, 17). Many available methods of supporting decisions in the case of uncertainty exist, utilizing data with uncertainty predictions and professional knowledge, which can in any case be used in risk management, especially in sensitive cases. This problematic has been thoroughly documented by Hans et al. in the form of a qualitative and quantitative method overview (18).

Pasman and Rogers (2020) (19) state, however, that predictive evaluation of risk is in fact connected to a great deal of uncertainty. The reason for this is incomplete danger identification and scenario definition, or the lack of a model and the lack of reliable data. Despite the expectation that wide-spread digitalisation would provide a source of “big” data, while the interpretation of analytics from this data would yield useful information, the so-called professional opinion and judgement remains a very useful source in the decision process when handling risk.

Stating the degree of risk for mobile sources is substantially more complicated. The risk evaluation of mobile sources of dangerous substances with regard to the endangerment of the environment and its select biotic components has until now been leaning only on qualitative and quantitative evaluation of the effects of a potential leakage of dangerous substances into the environment. An evaluation realized as such, however, is not sufficiently objective and its results are difficult to compare for individual biotic components. This aspect significantly complicates subsequent decision processes (20).

Crisis management is one of the most important parts of every organization, due to the unpredictability of people's behavior in times of crisis (21). Crisis is a non-routine, unexpected and sudden event, that threatens the primary goals of an organization. The stance that organizations facing a crisis take depends on who is held responsible for the given unfavorable situation. Crises can be differentiated from extraordinary events by the fact that they usually fall outside the general scope of organizations which they affect or which manage them (22–24). In addition to this, studies distinguish between crises and catastrophes on the basis of whether the cause is some internal failure of the organization (crisis) or an external event, which is outside the organisation's control (catastrophe) (25, 26).

This is why it is difficult to handle a crisis using conventional routines and procedures (27–29). Crises are by definition events which occur outside the general scope of interested organizations, hence it is hard to describe them and prepare for them ahead of time (30, 31). They usually occur due to various causes, like natural catastrophes (earthquakes, wildfires, dust storms and extreme colds), technical and technological catastrophes (process fires, explosions, and escapes of toxic substances) or humanitarian actions (acts of terrorism, sabotages, violence, and strikes). Regardless of the cause of crises, every organization must have a specific system of crisis management to minimize property damage and loss of human lives (27, 28). Decision-making has a large degree of importance in crisis management, since the people in charge are trying to pass a decision with minimal negative consequences in times of emergency (32).

An early detection of crisis indications substantially increases the options of saving property. Organizations must therefore investigate the causes of the problems which they encounter regularly in a complex manner, meaning they must be thoroughly analyzed, to determine whether they are the product of errors, or are in fact the product of inadequate and unsuccessful processes. Early and adequate resolution of basic causes of problems, hazards and incidents is the best method of crisis prevention. Crisis prevention must be based on a system in which risk management and crisis management will play a key part. From a managerial standpoint, certain crises could be avoided by monitoring internal and external information (sources of risks) and identifying problems (risks) in the early phases of their conception (33–35).

A system of crisis management usually contains four phases: mitigation, readiness, reaction, and renewal. A system of crisis management could be called an acknowledged system-of-systems, since there usually exists some type of control element, while elements of the system are independent to a significant degree, in areas which concern goals, financing and resources. Although most reactions require some basic capabilities, such as logistics, coordination and communication, the exact configuration will be different in individual crises. Furthermore, since threats and vulnerabilities change, the system of crisis management should be able to adapt and transform even in-between crises, so that it may stay relevant (36, 37).

Crises and risks, like natural disasters and accidents, represent a real and large threat to the lives and wellbeing of people, causing injuries, diseases, and deaths. In this context it is the goal of crisis management to lower the number of injuries and increase survival rates in stressful conditions. Crisis management is therefore also understood as an application of strategies designated for providing help to the interested parties with various skills or roles, whether it be from different organizations (medical unit, police, etc.) or the general public, to deal with unexpected negative events with the goal of limiting the severity of their effects (38).

Calliari et al. (39) claim that environmental damages caused by industrial pollution are the main cause for ecosystem degradation with a negative impact on human health, society, and economy. Damages to the environment can be caused by accidents or the slow onset of pollution during a longer period of time because of gradual contamination and accumulation of polluting substances. Industrial accidents with severe environmental damages are the result of inadequate risk prevention and/or dangerous substance management (39).

In recent memory, the direct evaluation of risks for the populace in response to emergencies – accidents (fires, explosions, leakage of dangerous substances) - has attracted large attention. Existing studies were predominantly concerned with negative consequences for life, and only then with the health of the population. Until now, great care went into evaluating direct risk for the population as a result of great accidents (fires, explosions, leakage of toxic substances). Much less care went into quantitative evaluation of the risks of consequences of great accidents for the environment. Although in current times, more and more studies with the goal of assessing risks and determining their negative consequences for people are being carried out (40–45). With an increasing number of vehicles on the roads, the number of car accidents increases as well. Various substances leak out of the vehicles in such accidents, and they can have a negative impact on the environment, which also affects the lives of people. To limit the spreading of these substances it is important to focus on the right reaction, which can prevent the spreading of the escaped substances. Substances which serve for capture of escaped dangerous substances, especially in car accidents are called sorbents.

Safety on the roads therefore directly influences the lives, health, property and environment of a society. The intensity of traffic rises year after year, and analysis of the traffic network together with questions about safety take center stage. The number of car accidents is increasing, and therefore ensuring a high level of safety on the roads is a necessary part of traffic (46). The release of dangerous materials caused by accidents during transport on the roads is inseparably linked to risks which have in recent years roused interest and concern of the public around the world. The immediate consequences of cargo vehicle accidents and of the transport of dangerous substances include abrupt contamination of soil and water with a following damaging of land and water ecosystems and subsequent economic losses (47). According other authors, a dangerous material is any substance, industrial or other waste that can due to its internal properties endanger human life and health, as well as damage the environment (48).

The occurrence of accidents involving the release of chemicals into the atmosphere and often causing deaths has forced environment security and government organizations to develop systems of real-time response based on accurate predictions and computer simulations. The influence of incidental release of toxic chemicals can be minimized by providing accurate information and by adequate management of large incident reaction teams (49). Accidents involving dangerous substances lead to consequences for the environment and the safety of nearby citizens. The severity of these accidents depends on the size and chemical properties of the leakage, as well as the susceptibility of the environment and the nearby presence of people (50).

The hazard of oil products and other dangerous substances is a looming threat that can lead not only to the loss of valuable products, but can also seriously damage the environment and ecosystems. There are various ways of combating these dangerous substances, one of which is the use of sorbents (51). Sorbents are substances which serve for capturing dangerous substances. According to their form we differentiate between loose and textile ones. In the case of car accidents, loose sorbents are utilized on the roads. On the basis of realized sorption, they are adsorption materials, since sorption occurs only on the surface of a given substance.

A part of the aforementioned challenges is also a quick, effective and safe capture of escaped dangerous substances, be it in an industrial area or during car accidents. For the given purpose, sorption resources are used, especially adsorption materials. Today, secondary processing materials are utilized. Car accidents, due to an increase in transport connected with the shift of the market into the online space have an increasing tendency to occur (52, 53). An appropriate application of adsorption materials creates the conditions for crisis aversion and lessens the negative impact on the environment. Sorbents are substances which serve for capturing dangerous substances. According to their form we differentiate between loose and textile ones. In the case of car accidents, loose sorbents are utilized on the roads. On the basis of realized sorption, they are adsorption materials, since sorption occurs only on the surface of a given substance. Versatile methods of removing organic pollutants as well as unwanted waste materials can cause direct environmental degradation. They can also be the reason for soil pollution as well as water contamination, as several authors point out in their research (54–58). Therefore, it is necessary to choose sorbents that are safe for the environment

Adsorbents are solid substances used in separation of components from fluid mixtures (liquids and gases). They can be divided according to chemical composition, structure (loose, textile), origin (natural, synthetic), sorption ability when dealing with polar on non-polar compounds and chemicals. Natural adsorbents (wood shavings, peat, sand, powdered sulfur, coal dust and others) have a lower absorption capacity in comparison to synthetic ones (59). Among the technologies, the adsorption was considered for the most used and safe protection of the environment. The concept of green adsorbents was introduced in terminologies, which means cheap materials originating from waste, i.e. agricultural waste as valuable adsorbents. An extensive list of different adsorbents from natural, industrial waste, agricultural bio products or modified materials have been applied to eliminate various contaminants from the water matrix. All adsorbents, by their intrinsic nature, have functional groups that play an important role in the adsorption of metal ions. In general, chemically modified adsorbents increase the surface area of the adsorbent and a higher unit of adsorption capacity than unmodified adsorbents (60–62).

**Figure 1:**
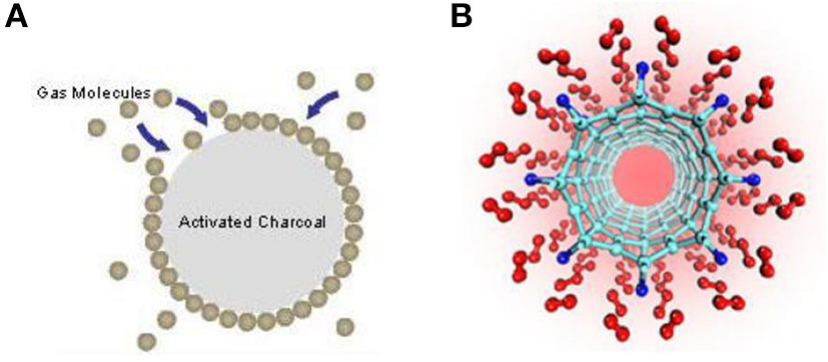
**(A,B)** An example of physical sorption on the surface of a solid sorbent in a single-molecule layer (48).

Synthetic adsorbents are manufactured as hydrocarbon pollution adsorbents. The so-called swollen perlites, also known as expanded perlites, form the largest group of these. The resultant material has an ability to absorb a liquid into its pores (Figure 1). The capture of a dangerous substance, which is most commonly oil pollution, happens in multiple simultaneous phases. These phases cannot be considered separately. They are absorption into the pores in the grains of the material and absorption into the pores in between the grains. The most effective capture is adsorption since it is defined by a stronger molecular bond of the capturing and captured substance. If we consider that adsorption does not exceed a layer of multiple molecules, or that it occurs in a single-molecule layer (Figure 1A), only a limited amount of liquid can be captured on the surface of the solid substance. The amount of liquid captured can be scaled with the increase of the surface area of solid particles, which scarcely occurs. By the influence of capillary forces, the absorption into the pores of the grains takes place (Figure 1B). This process seamlessly follows capillary condensation, which is in fact a phenomenon between adsorption and absorbency.

The essential difference is that capillary condensation fills in deep pores to the narrowest profile, which means it often fills them from the back. Absorbency into the pores occurs from the outside and the degree is given by the option of ventilation. The absorption into the pores between the grains is primarily the capture into the loose substance. Grains are not scattered, only touching one another. The liquid first enters the gaps between the grains and after soaking the walls of the grains it is pulled into the space between the grains by capillary forces, the process continues by soaking the walls of the pores inside individual grains and by absorbing the liquid into these pores (63). When evaluating their properties, emphasis is placed on “sorption ability”, which is the degree (ability) of the substance to absorb or imbibe an oil product or another undesirable substance. The quality of a sorption material is evaluated on the basis of sorption capacity.

The goal of the article is a presentation of risks connected with the leakage of dangerous substances. The acceptance of risk of dangerous substance leakage is followed by a description and analysis of the application of sorption resources for a quick and effective capture of the escaped substances, in particular oil and gasoline, as a reaction to the created event. The goal is at the same time the statement of the adsorption capacity of given adsorption materials.

**Figure 2:**
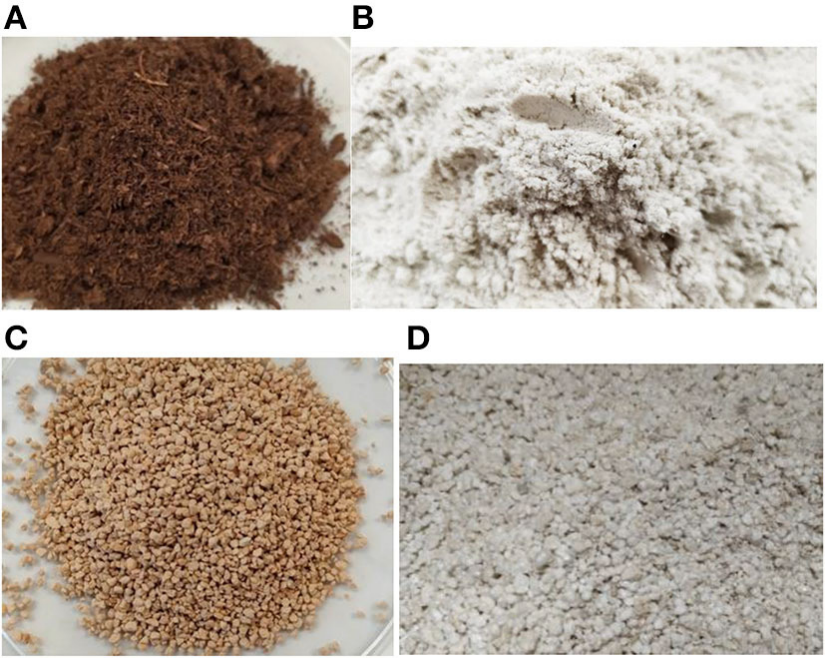
Illustration of the tested samples. **(A)** Sorb 1, **(B)** Sorb 2, **(C)** Sorb 3, **(D)** Sorb 4.

Sorb 1 is made from 100%-recycled powdered polyurethane (64) and it is a newly-developed product (Figure 2A). The sorbent is a product of secondary materials. Sorb 2 (Figure 2B) is a hydrophobic natural improvised sorbent – peat. The structure is loose; however, parts of wood can be found inside it. It belongs into a category of hydrophobic, natural, improvised sorbents. It is biologically degradable and hastens the biodegradation of the sorbate. It is good at binding hydrocarbon-based substances (65).

Sorb 3 (Figure 2C) is a mineral product. It is non-flammable and has a low dustiness (66). Sorb 4 (Figure 2D) is non-flammable, without a dangerous thermal decomposition, can float on the surface of water thanks to a hydrophobic coating on the surface. Its effectiveness lowers the effects of salts or dilute acids (Table 1). The creation of floating dust can occur during manipulation of the substance, it is good at sorption of substances even at high temperatures (67). Sorb 3 and Sorb 4 belong to a shared category of materials called perlites.

**Table 1:** Description of tested samples of loose sorption materials (64–67).

**Loose sorbent**	**Particle size (mm)**	**Chemical composition**	**Description**	**Package weight**
Sorb 1	0.12–2.0	100% polyurethane	A light-brown-colored powder	3 kg
Sorb	Inconsistent	Natural polymer	Brown color	6 kg
Sorb 3	0.8–2.0	85% SiO_2_ 6% Al_2_O_3_ 3% Fe_2_O_3_ 6% Others	Brown-red color	10 kg
Sorb 4	0.8–2.0	min. 66% SIO_2_ max. 18% Al_2_O_3_ max. 3% Fe_2_O_3_ max. 5% CaO + MgO max. 8% Na_2_O + K_2_O	Gray-white color	125 l

## Materials and methods

### Experimental samples – Loose adsorbents

Loose adsorbents are solid substances of various chemical composition. They are modified so that they have the largest possible surface area, which is useful particularly for the removal of thin layers of liquids on a large surface. Their drawback is dustiness. While using them, the environment becomes covered in a layer of filth (dust). On the other hand, they have long shelf lives, they are stored in bales.

For the purposes of this experiment, four samples commonly used in practice were utilized. One of them is a natural sorbent (peat) and three are synthetic ones with different chemical composition (Table 1).

All substances and sorption materials were conditioned for 24 hours at a temperature of 24°C and air humidity of 60% ± 5% according to ASTM F716-18, where it is stated to: “Suspend specimens in vapor space without contacting water for not less than 24 h prior to testing”.

### Adsorbed material – samples of polar and non-polar liquids

The research of sorption resources is realized according to established standards. The substrate on which the application of the sorbent is realized and the adsorption capacity is observed is oil. The discussed project is focused on the evaluation of the risk of a car accident. As a substrate material, oil, water, gasoline, and ethanol were chosen. The choice of the mentioned samples was intentional. Oil is a part of testing standards (68, 69), in our country, gasoline is the most commonly transported dangerous substance (70), water and gasoline were chosen as polar liquids, commonly used (Table 2).

**Table 2:** Description of substances chosen as adsorption substrates (71–73).

**Substrate sample**	**Polarity of liquid**	**Density (g.cm^−3^)**	**Flash point (°C)**	**Explosion limits**	**Viscosity (mm^2^.s^−1^)**
Oil	Non-polar	0.975 at 15°C	>80	X	4 at 40°C
Gasoline		0.750 at 15°C	−25	0.6–8 vol %	<1 at 37.8°C
Water	Polar	1.000	Non-flammable liquid		0.896 at 25°
Ethanol		1.040	14°C–closed cup	3.3–19	X

All experiments were realized with the same atmospheric conditions. All substrates were used for testing the sorption of the observed loose adsorption materials.

### Methodical procedure

The ASTM F716-18 (68) and ASTM F726-17 (74) standards have been developed for adsorbent performance and uptake capacity testing (A). Based on the Bazargan et al. (51) study of the option of stating the sorption capacity of given sorption materials, the normative procedure ASTM F716 – 18 (68) was chosen for the research.

**Figure 3:**
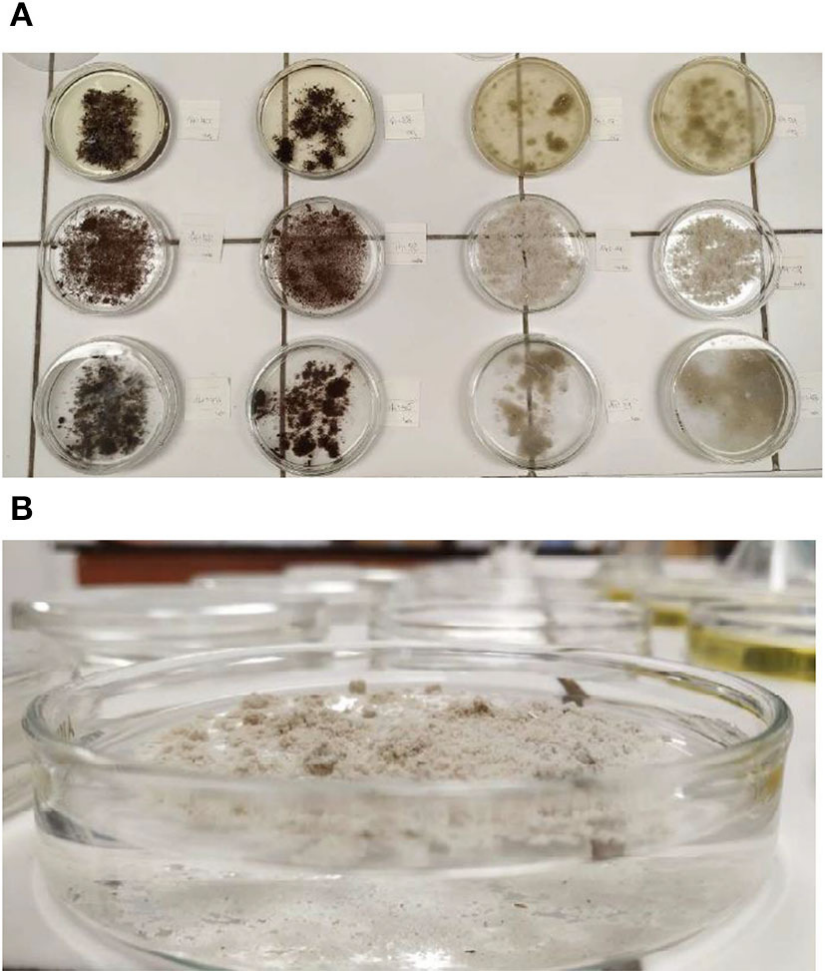
**(A)** Illustration of the experiment on the samples Sorb 1 and Sorb 2; **(B)** Observing the soakage of the sample Sorb 1.

The standard testing method of sorbents for testing the absorption in chemical and light hydrocarbon leaks observes the absorption ability of a loose sorbent to absorb the liquid in the container (Figure 2) during a two-hour period (Figure 3A). Three test samples of adsorbent were treated at room temperature 24°C (standard conditions are in the range of 21–26°C) in a closed vessel with 2.5 cm of water at the bottom. (Point 9.1 Standard ASTM F716-18. During the experiment, a loose sorbent was observed to float (Figure 3B) above the surface of the substance (this occurs if the absorbed substance has a greater density than the loose sorbent). In this case, the loose sorbent was weighed down by a weight placed perpendicular to the bottom of the container. The sorbent has not reached its maximum sorption capacity over the period of two hours, so the experiment was realized over the period of 24 h. The three adsorbent test speciemens were totally immersed in water at room temperature 24°C (standard 's condition is range 21–26°C) in a vessel with a minimum of 10 cm of water covering the absorbent for not less than 24 h prior to testing (Point 9.2 Standard ASTM F716-18). After the maximum absorption of the sorbent, the remaining sorbent has been filtered through a filter paper and the amount of the filtered unabsorbed substance was measured.

For the calculation of the sorption capacity, the following formula was used (1)


(1) $$A=\frac{m_{a}-m_{n}}{m_{n}}*100$$

Where, A is the adsorption capacity [%], ma the mass of the dry sorbent [g], mn the mass of the soaked sorbent [g].

## Results and discussion

### Results of evaluating loose sorbents according to ASTM F716-18

The observed loose sorbents are hydrophobic. The obtained values of A in the case of an adsorbed substance of water are low (Figure 3). It is impossible to compare the stated values to the values presented by the technical sheets of the mentioned products (Table 3).

**Table 3:** Comparison of experimentally acquired values of the adsorption capacity = adsorbent performance (capacity testing) (A) after 24 h of sorption and after 2 h of sorption on the water sample.

**Adsorbed substance: water**	**A per unit mass laden for 24 h**	**A per unit mass after 2 h**	**A given by the producer**	**Stated pH of the substrate after sorption**
Sorb 1	28	0.5	9	7.5
Sorb 2	19	3	27	5.3
Sorb 3	0	2.5	13	7.7
Sorb 4	12	1	18	7,97

**Figure 4:**
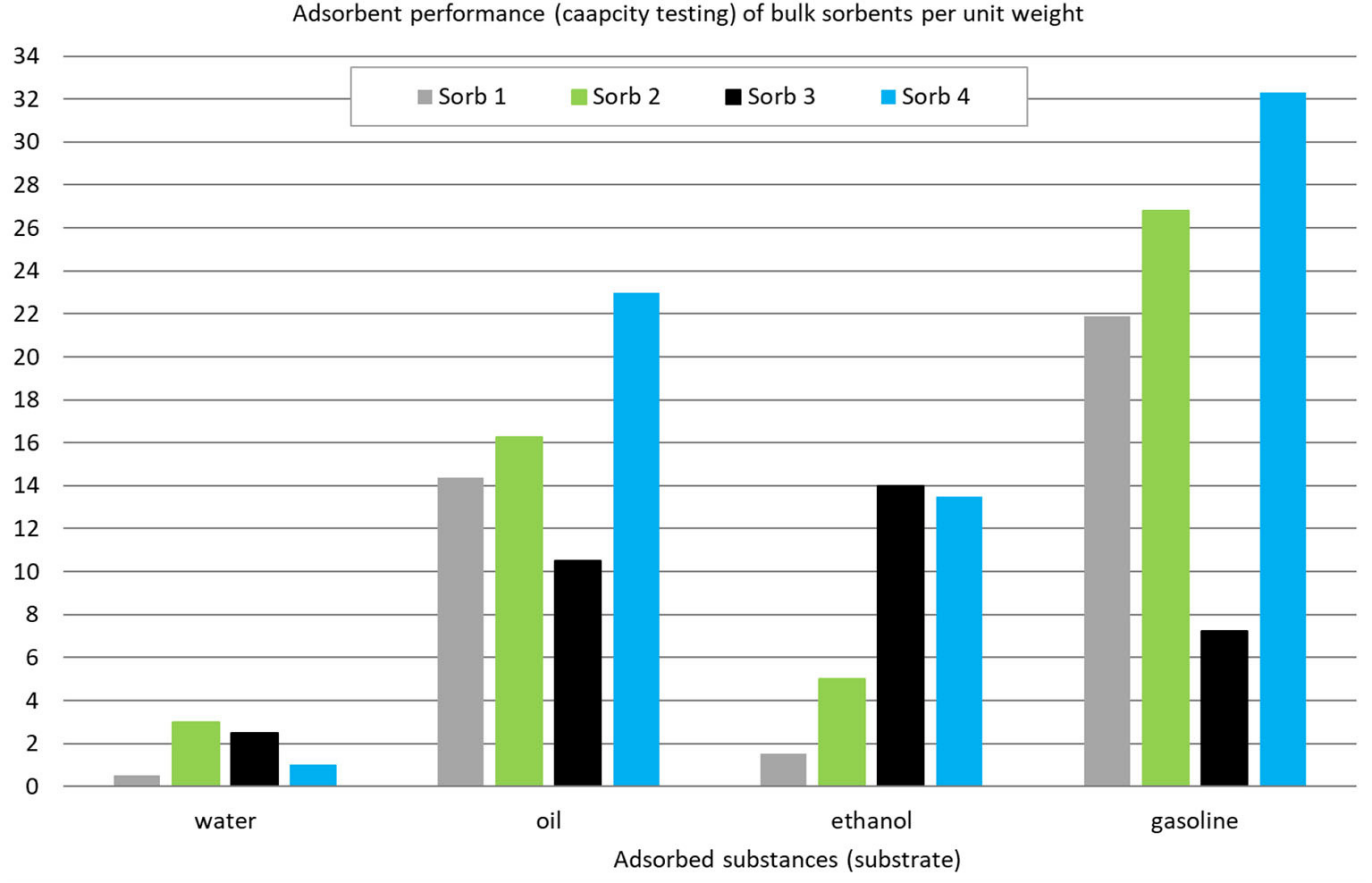
Comparison of adsorption capacity of loose sorbents per unit mass according to ASTM F716 – 18 (68) after 2 h.

The largest sorption capacity values were obtained using the Sorb 4 sample, which is based on 66% silica and 18% alumina. The stated ratio is important, because the Sorb 3 sample contains 85% silica and 6% alumina and its A values are significantly lower (Figure 4).

The reason for the improved performance of sorbent 2 (natural material) in water, of sorbent 3 in ethanol and of sorbent 4 in other mediums (oil and gasoline) can be found in the essence of the adsorption process. Adsorption is defined as an adhesion of chemical nature on the surface of particles it is an increase in the concentration of a substance on the dividing line of a solid and liquid layer due to the influence of surface forces (75). On the basis of established laws of physics, each liquid creates a layer on its surface whose molecules are being pulled into the center of the liquid by an attractive force. The pull on the molecules on the surface of the liquid into the center causes the surface to act as a thin elastic layer. The given layer is evaluated by the surface tension parameter. As an example surface tension (mN.m-1) at 20°C is 73 for water, 22 for alcohol (ethanol), 33 for olive oil, 27 for gasoline (not specified) / petrol (76). The surface tension data significantly affect the adsorption process. Water has the highest surface tension value and lowest adsorption values. The observed loose sorbents are hydrophobic. Differences occur in the experiment where sorbents were applied for 24 hours. The highest value was reached by the Sorb 1 sample due to a thorough utilization of the entire surface of the sample, which has not been achieved in the remaining samples. Sorb 2 is a natural peat, so its expected water intake as a natural material was high. The Sorb 3 sample behaves perfectly hydrophobically, but other samples absorbed certain portions of water due to a continuous exposure to water in atmospheric conditions. With a decrease in the surface tension value, adsorption capacity increases.

**Figure 5:**
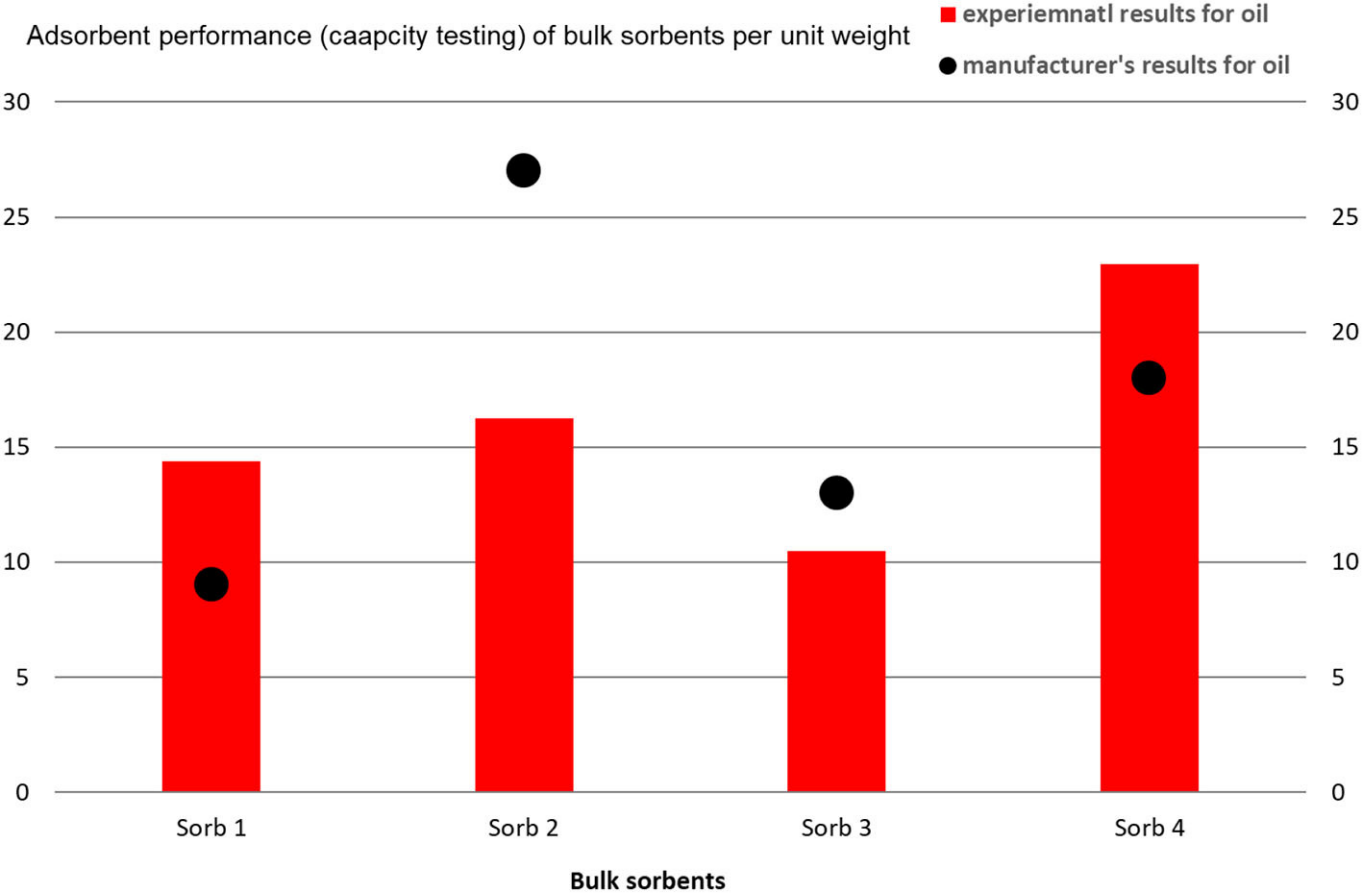
Mutual comparison of the AC of chosen loose sorbents for the sorption of oils, which are experimentally stated and obtained from the values in the manufacturer's technical documentation.

On the other hand, when evaluating A of oil sorption, values comparable to the manufacturer's values were obtained, in the case of Sorb 1 and Sorb 4, the experimental values were higher than stated by the manufacturer (Figure 5).

Conducting a detailed and quantitative analysis of the adsorption performance of different solid adsorbents against different dangerous substances escaped during car accidents with testing conditions, while also comparing the results to the referential values in norms is very difficult.

Norms used for stating the adsorption performance and the subsequently calculated sorption capacity do not state values of individual solid adsorbents for given dangerous substances. Manufacturers producing solid adsorbents state their own adsorption capacity values on safety data sheets, which apply to the motor oil referential substance. The given fact is accounted for on Figure 4. Some manufacturers also state the adsorption capacity values for water as a referential substance (Table 3).

Manufacturers have restricted the application of loose adsorption materials based on their composition. Hydrophobic ones are used for hydrocarbons (oils, organic gas, alcohols) and hydrophilic ones are used for capturing water and water solutions. In 2020, the European Chemicals Agency (ECHA) released the Guidance on the compilation of safety data sheets, where in the chapter 6.3 Methods and material for containment and cleaning up it states the application of adsorption materials as an appropriate method for removing an escaped substance or mixture. The given reality is supplemented by a note of incompleteness of the information given by ECHA (77).

Another parameter which evaluates the risk of dangerous substances leaking into the environment is mobility in soil. Mobility in soil is the potential of a substance or components of a mixture to get into subterranean water reserves or to escape from the place of leakage using natural forces after being released into the environment (article 12.4 in safety data sheet). The given parameter is only occasionally stated in SDS or in technical sheets of dangerous substances. The aforementioned parameter is found based on experimental data or probability models utilizing distribution constants (77).

### Environmental evaluation of loose sorbents

Another parameter that was observed according to ASTM F716-18 (74) is pH. The stated parameter evaluates the acidity or alkalinity of the environment. The pH standard is water, whose pH = 7 and creates a neutral environment. By the application of loose sorbents escaping dangerous substances are captured, while the mentioned application should also not alter the characteristics of the environment. The manufacturer themselves offers a relatively wide spectrum of pH for the Sorb 3 and Sorb 4 samples, which are based on silica and alumina.

**Figure 6:**
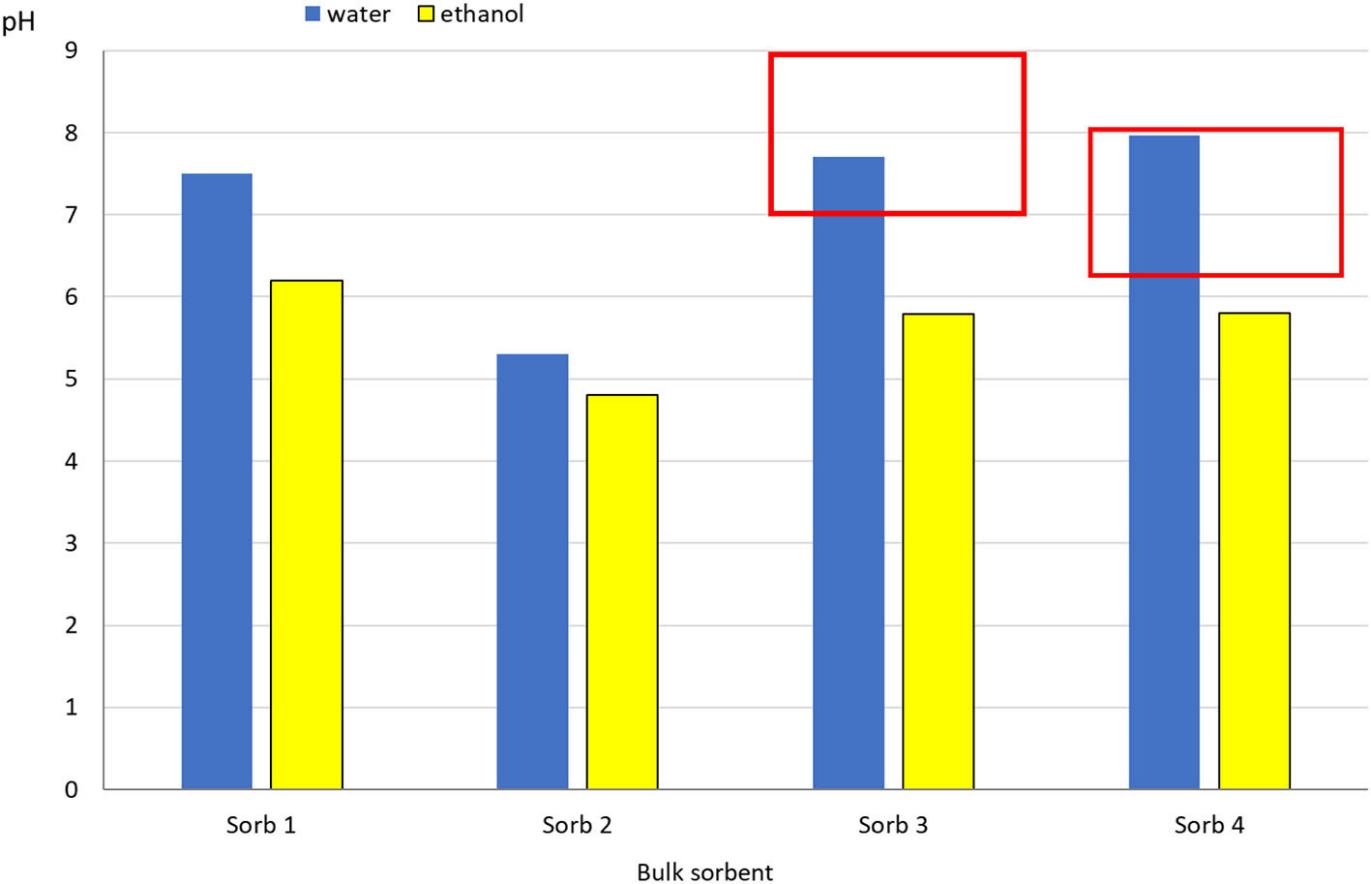
Comparison of pH of the resultant mixture of loose sorbents with water and alcohol. Legend: the red border represents the interval of pH given by the manufacturer.

They are sorbents on the same chemical basis (Table 1), the only difference between them being the percentual ratio of the given oxides. They are silicates, whose pH is around the neutral value. In the realm of the adsorbed substance of water, a match is observed. Worse values are obtained after the sorption of oil, where the pH falls into the acidic zone, in the Sorb 4 and Sorb 3 samples pH decreases below 6 (Figure 6). The Sorb 2 sample as a natural material at first glance provides a surprising result, where a decline occurs and the values are around pH = 5, which we would categorize as an acidic environment. However, available resources (78–80) state the possibility of utilizing the given natural material for the purposes of oxygenation of the environment.

The stated comment is meaningful in evaluating the given materials on the basis of environmental effect. Their application in the environment will not leave a dent in the acidity changes of the environment (except for Sorb 2), they do not disturb the biotope in which they were applied. The Sorb 2 manufacturer states biological degradability, which stems from its origin, but on the other hand the application of peat and the physical sorption of water and ethanol (polar liquids) results in the change of its character into the acidic region (an effect similar to the effect of acid rain).

Biological degradability is also mentioned in the technical documentation for Sorb 4. The application of Sorb 4 on polar liquids (water, ethanol) does not affect the acidity of the environment. The given statement can be extrapolated to Sorb 3 as well, since they have the same chemical composition, Sorb 4 being in fact richer in chemical components (Table 1).

Crisis management is focused on readiness, prevention, reaction, and the return to the initial state after the crisis (81). The experimental result offers a tool for the realization of steps in crisis management, especially in reaction. Reaction has a specific standing in the process of crisis management. For the reaction to be the best that it can be, it is necessary to be adequately prepared for various negative events. A correct reaction also lowers the negative impact on the environment and makes the return into the pre-crisis period easier. Based on the results it is necessary to equip emergency services with an adequate amount of sorbent in the event of dealing with the leakage of a dangerous substance. It is to the detriment of things that the results do not correspond to the data given by the manufacturer.

Road transport is connected with a plethora of incidents. Road safety is given its due of attention with regard to modern technologies, which should increase its quality (82, 83). Despite that it is currently impossible to prevent car accidents. Many accidents happen on the roads during which leakage of dangerous substances into the environment occurs. A significant deal of attention is therefore necessary to be given to reacting to the escape of dangerous substances during accidents. Multiple authors comment on the negative impact of dangerous substances on the environment and the need to resolve the problematic (84, 85).

Following the reaction, it is necessary to test various products and compare the real findings with technical sheets (86). In the event of a car accident connected with a leakage of certain dangerous substances into the environment it is possible to consider a proper usage of sorbents to capture the substances an effective reaction. The problematic of implementing sorbents is a part of many experiments, which aim to determine the correct sorbents or other substances to eliminate the negative impact of escaped substances on the environment (87–90). The data that we observed are beneficial in practice for emergency units, but also for crisis management or enterprises. The test results should find use in the choices of a buyer of a sorbent. The correct choice of sorbent can mitigate the financial costs for decontamination, capture of escaped dangerous substances and subsequent recultivation of the environment with the goal of diminishing the negative impact on the environment and the lives of the citizens living in the vicinity of the car accident.

## Conclusion

Crisis management concerns itself with reaction to a possible event, but an important task is also readiness. That was also the focus of the article, where we tested the adsorption capacity of sorbents. The tests themselves should be a part of preparation for negative occurrences, since in the event of a real leakage there is zero time or space to carry out a test. For this reason, the crisis managers and emergency services would have to depend only on the relevancy of the properties stated by the manufacturer and the technical sheet. Testing substances and comparing with available data is beneficial for a correct choice of sorbents used for the capture of an escaping dangerous substance in the event of a crisis.

The introduction of the experiments was devoted to water, where the testing method was validated and the hydrophobic property of loose sorbents with a sorption capacity equal to the one given by the manufacturer was confirmed. The values of sorption capacity for oil were significantly different to the ones given by the manufacturer. The highest sorption capacity values were achieved with the Sorb 4 sample, which is based on 66 % silica and 18 % alumina. The given ratio is important, because the Sorb 3 sample contains 85 % silica and 6 % alumina and its A values are significantly lower. The obtained data can make the choosing of a sorbent in the process of readying for crisis events easier. For emergency units they can also make a more correct choice of a kind and amount of sorbent possible.

## Data availability statement

The original contributions presented in the study are included in the article/supplementary material, further inquiries can be directed to the corresponding author.

## Author contributions

IM, JK, KB, and JR: conceptualization, writing–original draft preparation, validation, and investigation. IM, JK, and KB: methodology and writing–review and editing. IM: software, data curation, and supervision. JK and KB: formal analysis. JK, KB, and JR: resources. IM and JK: visualization. All authors contributed to the article and approved the submitted version.

## Funding

This work was supported by the Slovak Research and Development Agency under Contract No. APVV-20-0603 - Development of risk assessment tools for selected businesses and professions in the Slovak Republic in accordance with the EU requirements and by the Scientific Grant Agency of the Ministry of Education, Science, Research and Sport of the Slovak Republic—VEGA No. 1/0628/22 “The Security Research in Municipalities with Emphasis on the Citizens' Quality of Life”. This article was funded by the Grant System of UNIZA no. 16963: Research into the readiness of municipalities to deal with emergency events with an emphasis on the safety of the inhabitants.

## Conflict of interest

The authors declare that the research was conducted in the absence of any commercial or financial relationships that could be construed as a potential conflict of interest.

## Publisher's note

All claims expressed in this article are solely those of the authors and do not necessarily represent those of their affiliated organizations, or those of the publisher, the editors and the reviewers. Any product that may be evaluated in this article, or claim that may be made by its manufacturer, is not guaranteed or endorsed by the publisher.
